# Draft Genome Sequence of Environmental Isolate Acinetobacter nosocomialis U20-HoPe-S34-3 from Germany

**DOI:** 10.1128/MRA.00286-21

**Published:** 2021-05-27

**Authors:** Gottfried Wilharm

**Affiliations:** a Robert Koch Institute, Wernigerode Branch, Wernigerode, Germany; Indiana University, Bloomington

## Abstract

The draft genome sequence of Acinetobacter nosocomialis U20-HoPe-S34-3, isolated from soil sampled from the banks of the river Holtemme in Germany, is provided. The strain has an average nucleotide identity of 98.3% to the type strain of the species.

## ANNOUNCEMENT

The genus Acinetobacter comprises ubiquitously spread environmental species, as well as nosocomial pathogens with poorly defined natural habitats (1). Environmental isolates of the hospital pathogen Acinetobacter nosocomialis are extremely rare, with only a few confirmed reports available (2, 3). At the time of writing, the NCBI database included only A. nosocomialis genome sequences of human isolates or of unknown origin (https://www.ncbi.nlm.nih.gov/genome/browse/#!/prokaryotes/2169/). Here, we provide the genome sequence of a soil isolate of A. nosocomialis from Germany. The soil sample was collected from the waterside of the river Holtemme near Minsleben, Germany (51.863332 N, 10.830841 E), in October 2020. One gram of soil was resuspended in 10 ml of mineral medium (4) supplemented with 0.1% acetate as the sole source of carbon and energy and incubated at 37°C for 5 h with constant shaking. Subsequently, 100 μl of the suspension was spread onto Acinetobacter medium (CHROMagar, France) without the use of the CHROMagar multidrug-resistant (MDR) supplement and incubated for 24 h at 37°C. Reddish colonies tentatively identified as Acinetobacter baumannii were studied as detailed previously (5). While PCR analysis failed to detect the gene *bla*_OXA-51_-like, intrinsic to A. baumannii (6), in isolate U20-HoPe-S34-3, partial sequencing of the RNA polymerase subunit β gene *rpoB* (7) indicated that it belongs to the species (99.38% identity to the type strain of A. baumannii, compared with only 95.33% identity to the type strain of A. nosocomialis). To clarify its taxonomic position, the isolate was subjected to whole-genome sequencing. Genomic DNA was extracted with the MasterPure DNA purification kit (Epicentre) according to the manufacturer’s instructions from the pellet of 1 ml of an overnight culture grown at 37°C on a rotary shaker (160 rpm) in a 100-ml baffled flask with 10 ml liquid medium containing 10 g/liter tryptone, 5 g/liter yeast extract, and 5 g/liter NaCl. Shotgun libraries were generated using the Nextera XT DNA sample preparation kit and subjected to dual-index paired-end sequencing v3 (2 × 300 bp) on the Illumina MiSeq benchtop platform, yielding 2,782,230 reads in total. The raw sequence data quality was checked using FastQC v0.11.5 (8). Poor-quality and undersized reads were excluded using Trimmomatic v0.36 (9). Default parameters were used for all software unless otherwise specified. After further preprocessing (trimming at the 5′ and 3′ ends until the average quality was 30 in a window of 20 bases), the read files contained 480,119,357 bases in 2,208,078 reads with an average read length of 217 bases. Assembly with the Velvet v1.1.04 assembler integrated into Ridom SeqSphere v7.2.4 (10) using 1,895,833 of 2,208,078 reads yielded 133 contigs of at least 1,000 bases at 61-fold coverage on average and a total length of 3,937,225 bases (*N*_50_, 76,719 bases). The G+C content was 38.6%. The NCBI Prokaryotic Genome Annotation Pipeline (PGAP) v5.1 (11) identified 3,810 genes, of which 3,711 were protein coding sequences, 71 were RNA genes, and 28 were pseudogenes. Pairwise alignment of the 16S rRNA gene sequences from strain U20-HoPe-S34-3 and A. nosocomialis NIPH 2119^T^ revealed 99.93% identity, supporting the taxonomic classification of U20-HoPe-S34-3 as the species A. nosocomialis. The average nucleotide identity to A. nosocomialis NIPH 2119^T^ was 98.3%, as determined using autoMLST (12).

### Data availability.

This whole-genome shotgun project has been deposited at DDBJ/ENA/GenBank under the accession number JAFLQV000000000 (BioProject number PRJNA705907, BioSample number SAMN18106348, and SRA number SRX10250989).

## References

[1] Adewoyin MA, Okoh AI. 2018. The natural environment as a reservoir of pathogenic and non-pathogenic Acinetobacter species. Rev Environ Health 33:265–272. doi:10.1515/reveh-2017-0034. 29982240

[2] Carvalheira A, Silva J, Teixeira P. 2017. Lettuce and fruits as a source of multidrug resistant Acinetobacter spp. Food Microbiol 64:119–125. doi:10.1016/j.fm.2016.12.005. 28213015

[3] de Carvalho Girão VB, Martins N, Cacci LC, Coelho-Souza T, Nouér SA, Riley LW, Moreira BM. 2013. Dissemination of Acinetobacter nosocomialis clone among critically ill patients and the environment. J Clin Microbiol 51:2707–2709. doi:10.1128/JCM.00915-13. 23698521 PMC3719634

[4] Nemec A, Musílek M, Maixnerová M, De Baere T, van der Reijden TJK, Vaneechoutte M, Dijkshoorn L. 2009. Acinetobacter beijerinckii sp. nov. and Acinetobacter gyllenbergii sp. nov., haemolytic organisms isolated from humans. Int J Syst Evol Microbiol 59:118–124. doi:10.1099/ijs.0.001230-0. 19126734

[5] Łopińska A, Indykiewicz P, Skiebe E, Pfeifer Y, Trček J, Jerzak L, Minias P, Nowakowski J, Ledwoń M, Betleja J, Wilharm G. 2020. Low occurrence of Acinetobacter baumannii in gulls and songbirds. Pol J Microbiol 69:1–6. doi:10.33073/pjm-2020-011. PMC7256842 32162853

[6] Turton JF, Woodford N, Glover J, Yarde S, Kaufmann ME, Pitt TL. 2006. Identification of Acinetobacter baumannii by detection of the bla_OXA-51-like_ carbapenemase gene intrinsic to this species. J Clin Microbiol 44:2974–2976. doi:10.1128/JCM.01021-06. 16891520 PMC1594603

[7] Poppel MT, Skiebe E, Laue M, Bergmann H, Ebersberger I, Garn T, Fruth A, Baumgardt S, Busse H-J, Wilharm G. 2016. Acinetobacter equi sp. nov., isolated from horse faeces. Int J Syst Evol Microbiol 66:881–888. doi:10.1099/ijsem.0.000806. 26620413

[8] Andrews S. 2010. FastQC: a quality control tool for high throughput sequence data. Babraham Bioinformatics, Babraham Institute, Cambridge, United Kingdom.

[9] Bolger AM, Lohse M, Usadel B. 2014. Trimmomatic: a flexible trimmer for Illumina sequence data. Bioinformatics 30:2114–2120. doi:10.1093/bioinformatics/btu170. 24695404 PMC4103590

[10] Zerbino DR, Birney E. 2008. Velvet: algorithms for de novo short read assembly using de Bruijn graphs. Genome Res 18:821–829. doi:10.1101/gr.074492.107. 18349386 PMC2336801

[11] Tatusova T, DiCuccio M, Badretdin A, Chetvernin V, Nawrocki EP, Zaslavsky L, Lomsadze A, Pruitt KD, Borodovsky M, Ostell J. 2016. NCBI Prokaryotic Genome Annotation Pipeline. Nucleic Acids Res 44:6614–6624. doi:10.1093/nar/gkw569. 27342282 PMC5001611

[12] Alanjary M, Steinke K, Ziemert N. 2019. AutoMLST: an automated Web server for generating multi-locus species trees highlighting natural product potential. Nucleic Acids Res 47:W276–W282. doi:10.1093/nar/gkz282. 30997504 PMC6602446

